# Impact of Liability to Periodontitis on Glycemic Control and Type II Diabetes Risk: A Mendelian Randomization Study

**DOI:** 10.3389/fgene.2021.767577

**Published:** 2021-11-25

**Authors:** Parth D. Shah, C. M. Schooling, Luisa N. Borrell

**Affiliations:** Graduate School of Public Health and Health Policy, City University of New York, New York, NY, United States

**Keywords:** diabetes, glycemic control traits, periodontitis, epidemiology, mendelian randomization, causal analysis

## Abstract

While the association of periodontitis with Type II diabetes (T2DM) is well-established, the causal relationship remains uncertain. We examined the causal association of periodontitis with glycemic traits (HbA1c, fasting glucose, and fasting insulin) and T2DM using Mendelian randomization (MR) taking advantage of large genome-wide association studies of European and East Asian adults, i.e., the UK Biobank (*n* ≈ 350,000) (HbA1c), trans-ancestral MAGIC (HbA1c, fasting glucose, and insulin), and DIAMANTE (74,124 cases/824,006 controls), and AGEN for T2DM in Europeans and East Asians, respectively. Periodontitis was instrumented using single-nucleotide polymorphisms (SNPs), strongly and independently predicting liability to periodontitis in each ancestry group. SNP-specific Wald estimates were combined using inverse variance weighting. Sensitivity analyses were performed using the weighted median and MR-Egger with meta-analysis of MR estimates for Europeans and East Asians. Genetically instrumented liability to periodontitis was not associated with glycemic traits or T2DM in either ancestry or when ancestry specific estimates were meta-analyzed. Our findings do not support a causal association of liability to periodontitis with glycemic traits or T2DM. However, further research is required confirming these findings among other racial/ethnic groups, especially groups who carry a heavy burden of both periodontitis and T2DM.

## 1 Introduction

Type 2 diabetes (T2DM) is a leading cause of morbidity and mortality worldwide (World Health Organization, 2018; Zheng et al., 2018; Khan et al., 2020; Lin et al., 2020). About 9% of adults in the world now have diabetes mellitus, with 90% having T2DM (Zheng et al., 2018). Despite improved treatment and preventive measures, diabetes alone continues to be responsible for over a million deaths each year (World Health Organization, 2018; Khan et al., 2020). T2DM is a serious public health concern, as its burden is rising globally. Well-established risk factors include age, overweight, family history, and hypertension (American Heart Association, 2021). However, an emerging role of inflammation in glycemic control and pathogenesis of T2DM has been increasingly recognized as a means of improving prevention and control for this condition (Tsalamandris et al., 2019).

Similarly, periodontitis, one of the most common chronic inflammatory diseases of the gums and supporting structures of teeth (Papapanou et al., 2018), has long been considered a risk factor for T2DM (Preshaw and Taylor, 2011; Nazir, 2017; Liccardo et al., 2019). Periodontitis is caused by an immune response to oral bacteria (Cekici et al., 2014; Sudhakara et al., 2018) and has been associated with T2DM (Borgnakke et al., 2013; Stanko and Izakovicova, 2014; Wang T. F et al., 2014; Cao et al., 2019; Wu et al., 2020). The prevalence of periodontitis is between 20% and 50% in the general population (Nazir et al., 2020). According to the Global Burden of Disease Study, severe periodontitis was the 11th most prevalent condition in the world (GBD 2017 Disease and Injury Incidence and Prevalence Collaborators, 2017). Furthermore, some evidence suggests an association of periodontitis with poor glycemic control (Wang X et al., 2014; Cao et al., 2019). Therefore, determining whether the reported association of periodontitis with T2DM is causal is of paramount importance to population health.

A direct relationship of periodontitis with T2DM has been observed, possibly operating *via* insulin resistance (Mealey and Ocampo, 2007). Toxic substances, such as cysteine proteases, released by periodontal pathogenic bacteria may irritate endothelial cells and promote insulin resistance *via* endothelial cell inflammation and lipid deposition (Thorstensson et al., 1995; Grossi et al., 2004). In contrast, proinflammatory cytokines such as interleukin-6, C-reactive protein, and tumor necrosis factor-alpha may cause a downstream immune response, yielding antibodies reacting with endothelial cells and low-density lipoproteins, and promote insulin resistance (Preshaw and Taylor, 2011; Botero et al., 2016). However, it is unclear whether periodontitis is actually a causal target of intervention or a biomarker of the other exposures, which cause diabetes.

Previous studies have reported an association of periodontitis with diabetes (Borgnakke et al., 2013; Stanko and Izakovicova, 2014; Nazir, 2017; Liccardo et al., 2019; Wu et al., 2020). However, this well-established relationship of periodontitis with diabetes is primarily based on observational studies (Borgnakke et al., 2013; Graziani et al., 2018; Wu et al., 2020). Observational studies are subject to confounding by unknown and known factors (Fewell et al., 2007; Smith and Hemani, 2014), such as lifestyle, access to care, diet and nutrition, ill-health, and socioeconomic position, particularly as people living in poverty are often more vulnerable to T2DM (Hsu et al., 2012). Additionally, findings from clinical trials examining the effects of periodontal treatment on glycemic control in T2DM participants are mixed (Teshome and Yitayeh, 2017), with a number of trials showing no benefit of periodontal treatment in preventing or controlling T2DM (Engebretson et al., 2013; Wang T. F et al., 2014; Kara et al., 2015; Vergnes, 2015). Therefore, evidence on whether periodontitis is causally related to T2DM is lacking.

Given the lack of causal evidence for an association between periodontitis and T2DM, a potential solution is the comparison of the risk of disease in people with genetically different periodontal status, to take advantage of genetic randomization at conception. Mendelian randomization (MR), instrumental variable analysis with genetic instruments, provides an opportunity of obtaining unconfounded estimates from observational studies (Smith and Hemani, 2014; Burgess et al., 2020). In fact, a previous MR study (Yuan and Larsson, 2020) found a suggestive association of periodontitis with T2DM, but did not investigate replication or possible pathways *via* glycemic traits. To fill this research gap, we examined the causal relationship of genetic liability to periodontitis with glycemic traits [i.e., glycosylated hemoglobin (HbA1C), fasting glucose, and fasting insulin] and T2DM using large, available, suitable genome-wide association studies (GWAS) in both people of European and East Asian descent.

## 2 Materials and Methods

### 2.1 Overview of the Study Design

This study used MR to examine the causal relation of liability to periodontitis with T2DM and glycemic traits. MR uses genetic variants associated with a risk factor as instrumental variables (IVs), which can be tested for associations with disease outcomes (Smith and Ebrahim, 2003). This was a two-sample MR study utilizing summary genetic associations, where the single-nucleotide polymorphism (SNP)–exposure (periodontitis) and the SNP–outcomes (glycemic traits and T2DM) associations were largely taken from different studies. The main two-sample MR study was conducted in people of European descent, i.e., with instruments and exposures from different European descent populations. To repeat the analysis in people of East Asian descent, i.e., with instruments and exposures from East Asian populations, we used two-sample methods with overlapping samples.

### 2.2 Selection of Instrumental Variables for Periodontitis

For this study, we identified all SNPs from recent GWAS of clinically confirmed periodontitis in German, Dutch, or European American samples or from meta-analyses of these studies (Schaefer et al., 2010; Munz et al., 2017; Munz et al., 2019). Candidate SNPs were assessed for suitability against the assumptions required of a valid IV (Lawlor et al., 2008). Briefly, all IVs used must satisfy three assumptions (Lawlor et al., 2008). They must predict the exposure, only affect the outcome *via* affecting this exposure, and should not be associated with confounders of the exposure–outcome relationship.

This study was conducted utilizing the same SNPs used by previous studies examining the causal associations of periodontitis with hypertension (Czesnikiewicz-Guzik et al., 2019) and with cardiovascular diseases (Bell et al., 2020). Only independent (*r* ^2^ < 0.01) SNPs associated with liability to periodontitis at genome-wide significance (*p <* 5 × 10^–8^) were included. In cases where multiple SNPs were identified at the same locus, only the “lead” SNP (i.e., with the smallest *p*-value) was included. A total of five SNPs in *GLT6D1* (rs1537415), *LOC107984137* (rs729876), *MTND1P5* (rs16870060), *DEFA1A3* (rs2738058), and *SIGLEC5* (rs4284742) loci, previously associated with periodontitis (with *p <* 5 × 10^–8^) in GWAS, were identified for inclusion (Table 1) as IVs in our MR study (Munz et al., 2017; Munz et al., 2019).

**Table 1:** Characteristics of the SNPs predicting liability to periodontitis selected as instrumental variables for the Mendelian randomization analysis in Europeans and East Asians.

Population	SNP	locus	Gene	Effect allele	Non effect allele	Effect allele frequency	Beta	Standard error	Odds ratio	95% confidence interval
European	rs1537415	9q34.3	GLT6D1	C	G	0.41	0.4637	0.0797	1.59	(1.36, 1.86)
	rs4284742	19q13.41	SIGLEC5	G	A	0.76	0.2927	0.0521	1.34	(1.21, 1.48)
	rs2738058	8p23.1	DEFA1A3	T	C	0.43	0.2469	0.0415	1.28	(1.18, 1.39)
	rs16870060	8q22.3	MTND1P5	G	T	0.91	0.3075	0.0513	1.36	(1.23, 1.50)
	rs729876	16p13.12	LOC107984137	T	C	0.82	0.2151	0.0384	1.24	(1.15, 1.34)
East Asians	rs10737249	1	COLGALT2	C	T	0.91	0.2329	0.0457		
	rs192911809	12	SRRM4	A	G	0.01	1.2330	0.2525		
	rs117963472	18	RP11-161I6.2	G	A	0.02	0.4169	0.0903		
	rs118016840	21	DSCR8	G	C	0.01	0.6304	0.1247		
	rs9812091	3	CLSTN2	G	A	0.45	0.1218	0.0252		
	rs7756559	6	RP1-153P14.7	T	C	0.01	0.5933	0.1281		
	rs35067614	7	NECAP1P1	G	T	0.18	-0.1622	0.0333		

The *F*-statistics was obtained directly from a recent study (Bell et al., 2020) using the same SNPs, or calculated using an established approximation (Bowden et al., 2016). To assess potential pleiotropy, we obtained known genome-wide associations of each SNP used in this study from a curated phenotype to genotype cross-reference, MR-PheWAS (Millard et al., 2015). Replication was conducted using Biobank Japan (Nagai et al., 2017) to obtain East Asian-specific genetic predictors of liability to periodontitis (*r* > 0.01 using the East Asian reference panel and *p* < 5 × 10^−6^) in 3,219 cases and 209,234 controls. Power calculations were conducted using the approximation that sample size required for an MR study is the sample size for exposure on outcome divided by the *r* ^2^ for genetic instruments on exposure (Freeman et al., 2013).

### 2.3 Outcomes

Genetic associations with HbA1C were obtained from the UK Biobank summary statistics of (*n* = 361,194) people of White British ancestry, adjusted for sex, age, age^2^, sex*age, sex*age^2^, and 20 principal components for ancestry http://www.nealelab.is/uk-biobank/(Sudlow et al., 2015). Quality-controlled genetic associations with fasting glucose (mmol/L) (*n* = 46,613) and fasting insulin (pmol/L) (*n* = 43,750) in people of European descent without diabetes were obtained from MAGIC (Chen et al., 2021). Genetic associations were adjusted for body mass index (BMI), study-specific covariates, and principal components unless using a linear mixed model (Chen et al., 2021). DIAMANTE, the largest diabetes GWAS (74,124 T2DM cases and 824,006 controls), was used to obtain genetic associations with T2DM (Mahajan et al., 2018). Genetic associations with T2DM in Europeans were also obtained from FinnGen consortium (11,006 T2DM diabetes cases and 82,655 controls) (https://www.finngen.fi/fi). Similar genetic associations were also obtained from AGEN for East Asians. Specifically, trans-ancestral MAGIC GWAS (Chen et al., 2021) for East Asians was utilized to obtain genetic associations with HbA1C, fasting glucose, and fasting insulin in up to 33,307 people, and a GWAS of East Asians (Spracklen et al., 2020) including Biobank Japan (Imamura et al., 2016) to obtain genetic associations with T2DM (77,418 cases and 356,122 controls).

### 2.4 Statistical Analysis

SNPs were aligned on the same effect allele for exposure and outcome, also using effect allele frequency for palindromic SNPs. rs1537415 is palindromic with allele frequency 58%, so it could not be unequivocally aligned. Therefore, analyses were conducted with and without the palindromic SNP. In the primary analysis, SNP-specific Wald estimates (the estimate for SNP on outcome divided by the estimate for SNP on periodontitis) were combined using inverse variance weighting (IVW) with multiplicative random effects, which assumes balanced pleiotropy. Population-specific MR estimates for the same associations were combined using meta-analysis, with fixed effects when heterogeneity was low and random effects when heterogeneity was high (>30%).

### 2.5 Sensitivity Analysis

We used leave-out one plots to assess heterogeneity. We used weighted median (WM) and MR-Egger as sensitivity analysis (Burgess and Thompson, 2017). The WM may provide correct estimates even when the instruments, i.e., SNPs, are invalid for up to 50% of the weight. To test for horizontal pleiotropy, we used the intercept and 95% confidence interval (CI) of the MR-Egger regression line (Bowden et al., 2015). We assessed heterogeneity between the causal estimates of individual SNPs using Cochran’s *Q*-statistic for the IVW and MR-Egger methods. MR-Egger can be imprecise if the number of genetic instruments is low.

Statistical analyses were performed using ld_clump to select genetic instruments, and the MR package (Yavorska and Burgess, 2017) and metafor to combine estimates from different outcome studies in R (R Core Team, 2020). Two-sided *p*-values are reported throughout, with correction for multiple testing using a *p*-value of 0.05/2 = 0.025, given one disease outcome, diabetes, and one set of glycemic traits. This level of correction provides a balance between rigorous results and avoids false negatives. We used publicly available de-identified summary data without direct contact with study participants. Therefore, no ethical approval was required.

## 3 Results

### 3.1 Genetic Association With Periodontitis

Five uncorrelated SNPs, rs1537415, rs4284742, rs2738058, rs16870060, and rs729876, strongly associated with periodontitis (effect sizes of log transformed values) obtained from a GWAS (Munz et al., 2017; Munz et al., 2019) were used as instruments for making causal inferences about the role of periodontitis in glycemic traits and T2DM in Europeans (Table 1). Seven uncorrelated SNPs were obtained for liability to periodontitis in East Asians (Table 1); all had *F*-statistic >10 with average 23.6. Estimated *F*-statistics were >20 for Europeans, and therefore, hardly any weak-instrument bias would be expected (Bell et al., 2020). Consistent with a previous study (Bell et al., 2020), the proportion of variance explained in liability to periodontitis by each SNP was low. We also saw the highest proportion for rs1537415. This SNP explained 1.9% of the variance in periodontitis seen in a study (Bell et al., 2020). The other SNPs each explained approximately 0.3% of the phenotypic variance observed in their respective cohorts (Bell et al., 2020). At 80% power and 5% alpha, this study could approximately detect an odds ratio of 1.07 for diabetes and of 0.02 of an effect size for HbA1c.

**FIGURE 1:**
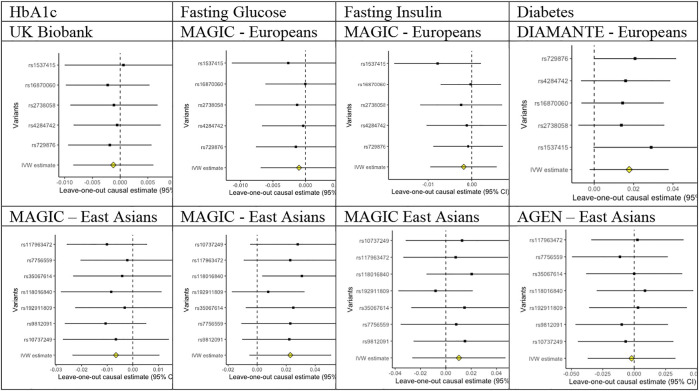
Leave-out-one plots for associations of liability to periodontitis with HbA1c, fasting glucose, fasting insulin, and diabetes in people of European and East Asian descent.

Of the five SNPs used to predict periodontitis in people of European descent, only rs4284742, rs2738058, and rs16870060 had known genome-wide associations with other phenotypes (Supplementary Table S1), most notably with immune cell counts for rs2738058, and with dental problems for rs4284742. Of the seven SNPs used to predict periodontitis in people of East Asian descent, only rs35067614 and rs10737249 had known genome-wide significant associations with other phenotypes (Supplementary Table S1), most notably with sitting height for rs10737249. As such, these associations most likely reflect vertical, and not horizontal, pleiotropy, and they were not excluded to preserve the phenotype. Correspondingly, the leave-out-one plots did not indicate heterogeneity (Figure 1).

### 3.2 Association of Liability to Periodontitis With Glycemic Traits

As shown in Table 2, all the analyses utilizing five SNPs or four SNPs in Europeans and seven SNPs in East Asians, respectively, genetically predicted that liability to periodontitis was unrelated to HbA1C, fasting glucose, and fasting insulin using IVW or WM. The MR-Egger intercepts were not different from the null value.

**TABLE 2:** Mendelian randomization estimates for liability to periodontitis on HbA1C, fasting glucose and fasting insulin, and T2DM using all five or four SNPs to predict periodontitis obtained from Europeans for the MR estimates in Europeans and seven SNPs to predict periodontitis in East Asians for the MR estimates in East Asians.

Outcome	Data source	Method	#SNPs	Beta	95% confidence interval	*p*-value	Cochran’s *Q*-statistic (*p*-value)	MR-Egger	
								Intercept *p*-value	*I* ^2^
HbA1C (effect size)	United Kingdom Biobank	IVW	5	−0.001	−0.008 to 0.006	0.74	1.38 (0.85)		0%
			4	0.001	−0.010 to 0.011	0.91	1.17 (0.76)		0%
		WM	5	−0.003	−0.011 to 0.006	0.56			
			4	−0.002	−0.014 to 0.011	0.81			
		MRE	5	−0.007	−0.032 to 0.018	0.58	1.14 (0.77)	0.63	71.0%
			4	−0.006	−0.089 to 0.077	0.89	1.14 (0.56)	0.88	14.9%
	MAGIC	IVW	7	−0.007	−0.024 to 0.010	0.45	7.65 (0.26)		21.6%
	East Asians	WM	7	−0.014	−0.033 to 0.006	0.18			
		MRE	7	−0.018	−0.047 to 0.012	0.24	6.55 (0.35)	0.36	0%
Fasting glucose (mmol/L)	MAGIC	IVW	5	−0.001	−0.007 to 0.005	0.74	1.81 (0.77)		0%
	Europeans		4	−0.003	−0.011 to 0.006	0.55	1.55 (0.67)		0%
		WM	5	0.0004	−0.007 to 0.007	0.91			
			4	−0.002	−0.013 to 0.008	0.71			
		MRE	5	−0.001	−0.021to 0.020	0.96	1.81 (0.62)	0.97	71.4%
			4	−0.043	-0.112 to 0.025	0.22	0.19 (0.91)	0.24	16.7%
	MAGIC	IVW	7	0.023	−0.005 to 0.051	0.11	9.39 (0.15)		36.1
	East Asians	WM	7	0.018	−0.013 to 0.050	0.26			
		MRE	7	0.037	−0.013 to 0.087	0.15	8.57 (0.13)	0.49	0%
Fasting insulin (pmol/L)	MAGIC	IVW	5	−0.002	−0.010 to 0.006	0.62	5.3 (0.26)		23.8%
	Europeans		4	−0.008	−0.018 to 0.002	0.12	2.8 (0.43)		0%
		WM	5	0.002	−0.006 to 0.010	0.66			
			4	−0.006	−0.019 to 0.007	0.34			
		MRE	5	0.013	−0.012 to 0.037	0.31	3.5 (0.32)	0.22	72.5%
			4	−0.023	−0.113 to 0.067	0.62	2.6 (0.27)	0.74	15.6%
	MAGIC	IVW	7	0.010	−0.027 to 0.047	0.58	11.7 (0.07)		48.7%
	East Asians	WM	7	0.004	−0.035 to 0.042	0.85			
		MRE	7	0.045	−0.013 to 0.102	0.13	8.21 (0.35)	0.14	0%
				**Odds Ratio**					
Diabetes	DIAMANTE	IVW	5	1.02	1.00 to 1.04	0.08	3.40 (0.49)		0%
	Europeans		4	1.03	1.00 to 1.06	0.053	2.36 (0.50)		0%
		WM	5	1.02	0.99 to 1.04	0.18			
			4	1.03	1.00 to 1.07	0.06			
		MRE	5	1.00	0.93 to 1.07	0.99	3.12 (0.37)	0.60	71.8%
			4	1.17	0.93 to 1.47	0.18	1.15 (0.56)	0.27	22.5%
	Finngen^a^	IVW	4	0.97	0.92 to 1.03	0.34	2.10 (0.55)		0%
	Europeans		3	0.96	0.89 to 1.03	0.27	1.72 (0.42)		0%
		WM	4	0.98	0.92 to 1.05	0.57			
			3	0.96	0.87 to 1.06	0.39			
		MRE	4	1.07	0.88 to 1.32	0.49	1.16 (0.56)	0.33	62.3%
			3	1.60	0.71 to 3.64	0.26	0.18 (0,67)	0.22	0%
	AGEN	IVW	7	1.00	0.96 to 1.03	0.91	5.38 (0.50)		0%
	East Asians	WM	7	0.98	0.94, to 1.03	0.45			
		MRE	7	0.97	0.92 to 1.03	0.32	4.01 (0.55)	0.24	0%
	Meta-analysis	Of IVW estimates	12	1.01	0.99 to 1.03	0.28			
			11	1.01	0.99 to 1.03	0.30			

a Rs729876 not available.

### 3.3 Association of Liability to Periodontitis With T2DM

Using all five SNPs as instruments for liability to periodontitis, there was no association of genetically predicted periodontitis with the risk of T2DM in DIAMANTE, or East Asians as presented in Table 2. This finding was also observed in FinnGenn regardless of whether four or three SNPS were used. Similar estimates were identified from the weighted median and MR-Egger sensitivity analyses. The MR-Egger intercept did not detect any directional pleiotropy for any outcome.

## 4 Discussion

We examined the causal association of periodontitis with glycemic traits (HbA1C, fasting glucose, and fasting insulin) and T2DM using an MR study. While the observational epidemiological findings suggest poor glycemic control (HbA1C) in patients with periodontitis (Taylor et al., 1996; Borgnakke et al., 2013), we found no reliable evidence for a causal association of liability to periodontitis with these glycemic traits or T2DM.

We identified and included five independent SNPs, associated with clinically defined periodontitis at a genome-wide significance. These SNPs have previously been used in another MR study (Bell et al., 2020). These five SNPs were all identified in cohort studies of aggressive periodontitis only, or of a mix of both aggressive and chronic periodontitis (Munz et al., 2017; Munz et al., 2019). Because chronic periodontitis is the predominant form of the disease, GWAS of solely aggressive periodontitis may not comprehensively capture the relevant phenotype or comprehensively identified genome-wide significant variants associated with the disease (Divaris et al., 2013; Teumer et al., 2013; Feng et al., 2014; Shaffer et al., 2014; Hong et al., 2015; Shimizu et al., 2015).

Confounding may be an explanation as to why these findings differ from observational studies (Fewell et al., 2007). Periodontitis and glycemic traits are both multifactorial in nature, representing individuals’ many years of exposure to risk factors. Observed associations could be confounded by other factors. For instance, smoking is an important risk factor for both periodontitis and glycemic traits (Genco and Borgnakke, 2013; U.S. Food & Drug Administration, 2020). Other possible confounders include age, obesity, and socioeconomic position (Genco and Borgnakke, 2013; U.S. Food & Drug Administration, 2020; American Heart Association, 2021). Obesity, a known contributor to the risk of increased HbA1C, fasting glucose, and fasting insulin (American Heart Association, 2021), has also been found to affect the development of periodontitis (Genco and Borgnakke, 2013). However, our study did not find a causal relationship of periodontitis with glycemic traits. Therefore, there is no evidence for prioritizing treatment of periodontitis for the control of glycemic traits.

A previous MR study reported an association of periodontitis with blood pressure, even though the association accounted for only a very small portion of blood pressure variation (Czesnikiewicz-Guzik et al., 2019). Hypertension is a well-established risk factor for T2DM (American Heart Association, 2021). It is likely that the contribution of periodontitis to hypertension might be small, and as a result, we did not observe evidence of a causal association of periodontitis with T2DM. This finding is inconsistent with the possible association suggested by a previous MR study (Yuan and Larsson, 2020) examining a series of risk factors for type 2 diabetes. However, the previous study did not investigate replication or possible pathways *via* glycemic traits.

Our study had a number of limitations worth discussing. The variance explained by the five SNPs used was relatively low, because the selected variants are associated with the aggressive form of periodontitis. However, the *F*-statistics were larger than 10. Furthermore, we selected the lead SNP from each locus, not on the basis of a linkage disequilibrium threshold as it is usually done. It is therefore plausible that more instruments might have given more conclusive findings. However, we chose variants drawn from three separate GWAS with unavailable full summary statistics. Our findings should be considered in the context of the underlying assumptions and limitations of MR. First, estimates derived from MR analyses generally represent the effect of cumulative exposure to a risk factor, and thus, may not correspond exactly to the expected outcome of a particular exposure, with regards to duration, timing, and mechanism. The genetic associations with fasting glucose and insulin were adjusted for BMI (Chen et al., 2021), which could bias as a heritable covariate (Aschard et al., 2015), possibly towards the null. However, the associations for liability to periodontitis with fasting glucose and fasting insulin were similar to those for HbA1c. Also, MR studies are open to survival bias. We could not totally exclude the possibility that the null results might be explained in part by prior deaths from sequalae of T2DM and from other conditions that preclude people living long enough to have periodontitis.

This study considers mostly people of European and East Asian descent because of the availability of suitable GWAS although separate samples for exposure and outcome were only available for people of European descent. However, bias from using overlapping samples may not be as much of a concern as has been thought (Minelli et al., 2021). Periodontitis may manifest differently in different populations, but there is no evidence of population-specific mechanisms. Moreover, using publicly available data precludes subgroup analysis by age, sex, and baseline periodontal status. Finally, we were not able to conduct a bi-directional MR study giving associations of glycemic traits or T2DM with periodontitis because the relevant GWAS are too small for meaningful analysis or are not publicly available. Previous evidence concerning a bi-directional association of diabetes with periodontitis is limited (Taylor and Borgnakke, 2008; Taylor et al., 1996).

Despite these limitations, our study had several strengths. We conducted the analyses using five liability to periodontitis SNPs as well as four such SNPs, and use multiple sensitivity analyses to confirm our results. We did not identify a causal association of periodontitis with glycemic traits or T2DM using IVW, WM, or MR-Egger methods. The consistency of these findings with both analyses using five SNPs and four SNPs, and the large sample sizes used for both outcomes suggest robustness. We also included analysis for East Asians and replicated based on genetic instruments from a similar population.

The GWAS of liability of periodontitis to date failed to identify consistent SNPs (Divaris et al., 2013; Teumer et al., 2013; Feng et al., 2014; Shaffer et al., 2014; Hong et al., 2015; Shimizu et al., 2015). The reason for divergent SNPs identified in periodontitis GWAS could be due to inconsistent definitions of periodontitis that were used in different studies (Sun et al., 2020). To confirm the causal effect of periodontitis on risk of glycemic traits and T2DM, stronger instruments for periodontitis derived from large-scale GWAS with a consistent definition of periodontitis (Caton et al., 2018) are warranted. It is also of interest to perform similar studies in a wider range of racial/ethnic populations.

In conclusion, we found that liability to periodontitis was associated neither with glycemic traits nor with T2DM. While these findings are intriguing, MR analysis provides strong evidence of a non-causal association of periodontitis with glycemic traits and T2DM risk. However, more research is required to confirm these findings among other racial/ethnic groups, especially groups who seem to carry a heavy burden of both periodontitis and T2DM.

## Data Availability

Publicly available datasets were analyzed in this study. These data can be found here: United Kingdom Biobank Summary Statistics: http://www.nealelab.is/uk-biobank/; MAGIC https://magicinvestigators.org; DIAMANTE https://magicinvestigators.org; AGEN https://hugeamp.org/downloads.html#T2D; FinnGenn https://www.finngen.fi/fi; and Biobank Japan http://jenger.riken.jp/result.
